# [^89^Zr]Zr-cetuximab PET/CT as biomarker for cetuximab monotherapy in patients with RAS wild-type advanced colorectal cancer

**DOI:** 10.1007/s00259-019-04555-6

**Published:** 2019-11-09

**Authors:** E. J. van Helden, S. G. Elias, S. L. Gerritse, S. C. van Es, E. Boon, M. C. Huisman, N. C. T. van Grieken, H. Dekker, G. A. M. S. van Dongen, D. J. Vugts, R. Boellaard, C. M. L. van Herpen, E. G. E. de Vries, W. J. G. Oyen, A. H. Brouwers, H. M. W. Verheul, O. S. Hoekstra, C. W. Menke-van der Houven van Oordt

**Affiliations:** 1 Medical Oncology, Cancer Center Amsterdam, Amsterdam UMC, Location VU Medical Center, Amsterdam, The Netherlands; 2 Julius Center for Health Sciences and Primary Care, University Medical Center Utrecht, Utrecht University, Utrecht, The Netherlands; 3 Medical Oncology, University Medical Center Groningen, University of Groningen, Groningen, The Netherlands; 4 Medical Oncology, Radboud University Medical Center, Nijmegen, The Netherlands; 5 Radiology and Nuclear Medicine, Cancer Center Amsterdam, Amsterdam UMC, Location VU Medical Center, Amsterdam, The Netherlands; 6 Pathology, Cancer Center Amsterdam, Amsterdam UMC, Location VU Medical Center, Amsterdam, The Netherlands; 7 Radiology and Nuclear Medicine, Radboud University Medical Center, Nijmegen, The Netherlands; 8 The Institute of Cancer Research and The Royal Marsden Hospital, London, UK; 9 Nuclear Medicine and Molecular Imaging, University Medical Center Groningen, University of Groningen, Groningen, The Netherlands

**Keywords:** Colorectal cancer, Targeted therapy, Cetuximab, Imaging biomarker, PET/CT

## Abstract

**Purpose:**

One-third of patients with *RAS* wild-type mCRC do not benefit from anti-EGFR monoclonal antibodies. This might be a result of variable pharmacokinetics and insufficient tumor targeting. We evaluated cetuximab tumor accumulation on [^89^Zr]Zr-cetuximab PET/CT as a potential predictive biomarker and determinant for an escalating dosing strategy.

**Patients and methods:**

PET/CT imaging of [^89^Zr]Zr-cetuximab (37 MBq/10 mg) after a therapeutic pre-dose (500 mg/m^2^ ≤ 2 h) cetuximab was performed at the start of treatment. Patients without visual tumor uptake underwent dose escalation and a subsequent [^89^Zr]Zr-cetuximab PET/CT. Treatment benefit was defined as stable disease or response on CT scan evaluation after 8 weeks.

**Results:**

Visual tumor uptake on [^89^Zr]Zr-cetuximab PET/CT was observed in 66% of 35 patients. There was no relationship between PET positivity and treatment benefit (52% versus 80% for PET-negative, *P* = 0.16), progression-free survival (3.6 versus 5.7 months, *P* = 0.15), or overall survival (7.1 versus 9.4 months, *P* = 0.29). However, in 67% of PET-negative patients, cetuximab dose escalation (750–1250 mg/m^2^) was applied, potentially influencing outcome in this group. None of the second [^89^Zr]Zr-cetuximab PET/CT was positive. Eighty percent of patients without visual tumor uptake had treatment benefit, making [^89^Zr]Zr-cetuximab PET/CT unsuitable as a predictive biomarker. Tumor SUV_peak_ did not correlate to changes in tumor size on CT (*P* = 0.23), treatment benefit, nor progression-free survival. Cetuximab pharmacokinetics were not related to treatment benefit. *BRAF* mutations, right-sidedness, and low sEGFR were correlated with intrinsic resistance to cetuximab.

**Conclusion:**

Tumor uptake on [^89^Zr]Zr-cetuximab PET/CT failed to predict treatment benefit in patients with *RAS* wild-type mCRC receiving cetuximab monotherapy. *BRAF* mutations, right-sidedness, and low sEGFR correlated with intrinsic resistance to cetuximab.

## Introduction

Cetuximab is a chimeric human murine immunoglobulin G1 monoclonal antibody (mAb) directed towards the extracellular domain of the epidermal growth factor receptor (EGFR). Cetuximab binds to EGFR and prevents phosphorylation of the downstream signaling effectors, such as the mitogen-activated protein kinase and PI3K/AKT/mTOR pathway, responsible for cell proliferation and growth, migration, adhesion, and inhibition of apoptosis. Additionally, cetuximab induces receptor downregulation and antibody-dependent cellular cytotoxicity [1]. The drug was registered as treatment for patients with metastatic colorectal cancer (mCRC) in 2008. Remarkably, tumor EGFR protein expression has no direct relationship with drug efficacy [2]. However, an activating mutation in one of the *RAS* proteins (*NRAS* and *KRAS* exon 2-4) is predictive for primary resistance to anti-EGFR mAbs [3]. Also, recent meta-analyses demonstrated that patients with a *BRAF V600E*–mutated tumor do not benefit from the addition of anti-EGFR mAbs [4, 5]. Additionally, patients with right-sided CRC derive less benefit from anti-EGFR mAbs and are also currently excluded for anti-EGFR mAbs in Europe [6, 7].

However, even when selecting patients based on *RAS* and *BRAF* mutation status, still one-third of patients do not benefit from anti-EGFR treatment. A potential explanation may be the highly variable pharmacokinetics (PK) of cetuximab. Clinical studies demonstrated an association between higher global clearance and lower trough levels of cetuximab and a shorter progression-free survival (PFS) [8, 9]. A lower blood concentration could result in reduced intratumoral accumulation of cetuximab and thereby reduced treatment efficacy. Indeed, in preclinical studies, tumor uptake of zirconium-89 labeled cetuximab ([^89^Zr]Zr-cetuximab) in xenograft bearing nude mice was not only dependent on tumor EGFR expression but also on pharmacokinetic and dynamic mechanisms [10]. Previously, we described that tumor accumulation of cetuximab could be assessed by using [^89^Zr]Zr-cetuximab positron emission tomography/computed tomography ([PET/CT) and found that tumor uptake varied between patients with mCRC, and that absence of [^89^Zr]Zr-cetuximab uptake could be related with less treatment benefit, suggesting that visible [^89^Zr]Zr-cetuximab uptake may be a prerequisite for efficacy [11].

Therefore, the primary aims of this study were to evaluate if patients without tumor uptake on [^89^Zr]Zr-cetuximab PET/CT with standard therapeutic pre-dose would have uptake upon dose escalation, and whether uptake would relate to treatment benefit. In addition, we assessed other factors that might contribute to drug accumulation and treatment efficacy, such as intra-patient variability in PK, tumor EGFR expression [10], plasma soluble EGFR (sEGFR) concentration [12], tumor perfusion on [^15^O]H_2_O PET/CT, and metabolic tumor activity on [^18^F]FDG PET/CT.

## Methods

### Population

Patients were eligible for inclusion if they had unresectable *RAS* wild-type mCRC, refractory to or contra-indications for standard chemotherapy (fluoropyrimidine, irinotecan, and oxaliplatin) and were naïve for anti-EGFR mAbs. Eligible patients were 18 years or older and had ≥ 1 extra-hepatic metastasis ≥ 20 mm diameter (tumor volume ≥ 4 mL to circumvent partial volume effects which hamper quantification of PET tracer uptake) [13]. Hepatic metastases cannot be evaluated due to intense uptake of [^89^Zr]Zr-cetuximab in healthy liver tissue [11]. Other inclusion criteria included ECOG performance status ≤ 2 and adequate renal and liver functions. At the time of this study (recruitment 2014–2017), patients with right-sided and *BRAF V600E*–mutated CRC were still eligible for cetuximab monotherapy. The study was performed at the Amsterdam University Medical Center location VUmc, Radboud University Medical Center, and University Medical Center Groningen, the Netherlands. The central Medical Research Ethics Committee of the Amsterdam University Medical Center location VUmc approved the study. All patients gave written informed consent prior to any study procedure. Follow-up was done until February 2019.

### Study

**Fig. 1:**
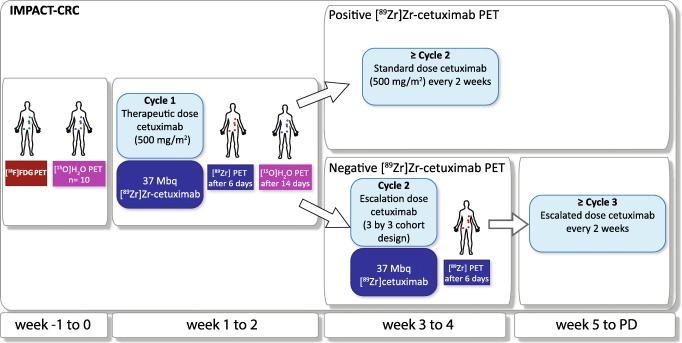
Study design of the IMPACT-CRC study. Before the first cetuximab cycle a [^18^F]FDG PET/CT was performed, and in a subgroup a [^15^O]H_2_O PET/CT was performed. Directly after administration of the first treatment dose of cetuximab (500 mg/m^2^), 37 MBq/10 mg [^89^Zr]Zr-cetuximab was injected and 6 days p.i. a PET/CT was performed. If there was visual uptake in at least one extra-hepatic lesion, the [^89^Zr]Zr-cetuximab PET/CT was considered positive and patients were treated with the standard dose every other week. In case of a negative PET/CT scan, cetuximab dose was escalated in a 3 × 3 cohort design and [^89^Zr]Zr-cetuximab PET/CT was repeated. These patients were treated with the escalated dose every other week. In an additional four patients an extra [^89^Zr]Zr-cetuximab PET/CT with low pre-dose (100 mg unlabeled cetuximab) was performed 14 days prior to treatment cycle 1 to explore the possible occurrence of saturation at a 500 mg/m^2^ dose (not indicated in this figure). These 4 patients underwent a second [^89^Zr]Zr-cetuximab tracer administration and [^89^Zr]Zr-cetuximab PET/CT after administration of the first treatment dose and continued with standard dose cetuximab every other week

The IMPACT-CRC is a phase I–II multicenter image-guided dose escalation study (NCT02117466; Fig. 1); here we report part 1. Patients underwent [^89^Zr]Zr-cetuximab PET/CT with the first therapeutic dose (500 mg/m^2^) as pre-dose within 2 h prior to injection of 37 MBq/10 mg of [^89^Zr]Zr-cetuximab. Three nuclear medicine physicians assessed tumor uptake independently (OSH, ABR, WOY). Only if ≥ 2 nuclear medicine physicians scored ≥ 1 extra-hepatic lesion as visually positive (enhanced uptake compared with local background), the PET scan was considered positive, and patients continued with the standard treatment dose (500 mg/m^2^) in a 2-week schedule. If the [^89^Zr]Zr-cetuximab PET/CT was negative, an escalated therapeutic dose (≤ 50% increase of cetuximab in each cohort) in a 3 × 3 cohort design was administered and another [^89^Zr]Zr-cetuximab PET/CT was performed subsequently after a second [^89^Zr]Zr-cetuximab tracer administration. These patients continued with the higher cetuximab dose in a 2-week treatment schedule. For all patients, treatment was continued until progressive disease, death, severe toxicity, or refusal of the patient. In case of toxicity, dose reductions were allowed as per standard clinical practice.

In an additional four patients a PET/CT of [^89^Zr]Zr-cetuximab (37 MBq/10 mg) was performed at a low pre-dose of 100 mg unlabeled cetuximab 7 days before start of therapeutic cetuximab treatment, to study the possibility of saturation effects by the therapeutic pre-dose (exploratory low pre-dose part of the study). Hereafter, these patients underwent a second tracer administration and another [^89^Zr]Zr-cetuximab PET/CT as above at the start of treatment (500 mg/m^2^ cetuximab). OSH assessed the PET scans in this exploratory phase.

Evaluation of study (imaging) data was supported by online data analysis software from an IT research infrastructure project, CTMM-TraIT, to allow multicenter blinded PET reviewing [14].

The data that support the findings of this study are available on request from the corresponding author. The data are not publicly available due to privacy or ethical restrictions.

Clinical outcome was defined in 3 measures. First, treatment benefit was defined as patients without progressive disease (stable disease, partial response, complete response) at first CT response evaluation according to RECISTv1.1 after 8 weeks of treatment [15]. Second, PFS was defined as the period between the first treatment cycle and progressive disease, or death of any cause, whichever came first. To evaluate PFS, patients underwent a CT every 8 weeks during treatment. Third, overall survival (OS) was defined as the period between the first treatment cycle and death of any cause.

### [^89^Zr]Zr-cetuximab PET/CT

[^89^Zr]Zr-cetuximab production complied with Good Manufacturing Practice [11]. Within 2 h after unlabeled cetuximab administration (dose depended on escalation phase), 10 mg of [^89^Zr]Zr-cetuximab (37 ± 1 MBq) was injected (for quantitative PET measurements, residual activity in the syringe was subtracted). Whole-body PET/CT (mid-femur-skull vertex, 5 min per bed position) and low-dose CT were acquired 6 days post injection (p.i.), for optimal tumor to background ratio based on a previous [^89^Zr]Zr-cetuximab PET/CT study with multiple time points [11]. All PET scanners were cross-calibrated and all PET data were reconstructed equally, normalized, and corrected for scattered and random coincidences, attenuation (based on CT scan), and decay [16].

Tumor lesion volumes of interest (VOIs) were delineated on the low-dose CT, which were then placed on PET images and corrected if necessary. Uptake in the tumor VOI was expressed in standardized uptake value (SUV), defined as the voxel activity divided by the injected dose (ID), and divided by body weight. SUV_mean_ (mean SUV of all voxels in the VOI) and SUV_peak_ (SUV_mean_ of a 12-mm-diameter sphere in part of the VOI with the highest uptake) were evaluated.

For [^89^Zr]Zr-cetuximab PK, image-derived blood activity was evaluated in a VOI of 5 voxels on 5 consecutive planes in the aortic arch. [^89^Zr]Zr-cetuximab concentration in plasma and whole blood was measured at the time of PET (6 days p.i.). Blood and plasma (centrifuged 4000 rpm for 5 min) samples were pipetted (0.5 mL) and weighted in duplicate. Radioactivity was measured using a single-well gamma counter (PerkinElmer) and decay corrected.

### [^18^F]FDG PET/CT

Within 2 weeks before the first treatment with cetuximab, [^18^F]FDG PET/CT was performed according to the EANM guidelines [16]. Briefly, patients fasted 6 h before tracer injection, with a target serum glucose of ≤ 7 mmol/L. Mid-femur-skull vertex PET/CT was performed 60 min (± 5 min) after injection of [^18^F]FDG (3-4 MBq/kg). PET scanners were cross-calibrated and all PET data were normalized and corrected for scattered and random coincidences, attenuation, and decay [16]. Tumor VOIs were semiautomatically delineated on PET images using a 50% SUV_peak_ threshold corrected for local background (≤ SUV 4).

### [^15^O]H_2_O PET/CT

In a subgroup of 10 consenting consecutive patients at the Amsterdam UMC location VUmc that had ≥ 1 intra-thoracic/upper abdomen tumor lesions, a [^15^O]H_2_O PET/CT (370 MBq) was performed to determine tumor perfusion at baseline (≤ 2 weeks before cetuximab cycle 1) and on-treatment (before cetuximab cycle 2). Tumor perfusion was calculated by modeling the tumor time-activity curve (TAC) using a VOI in the thoracic aorta as input function [17]. Perfusion was expressed in K1 (mL/cm^3^/min), which is the exchange of radiotracer from blood to tissue compartment. K2 (/min) is the exchange from tissue to blood compartment. The volume of distribution (Vt) is defined as K1/K2 (Fig. S1).

### EGFR and Ki67 immunohistochemistry

In pre-treatment formalin-fixed paraffin-embedded tumor tissue, EGFR and Ki67 (proliferation marker) were evaluated with immunohistochemistry (IHC). Staining of the two proteins was done using murine antibodies directed towards EGFR (Leica Biosystems, Nussloch, Germany, clone EGFR25, catalog number NCL-EGFR-384-L-CE) or Ki67 (Dako, Glostrup, Denmark, clone MIB1, catalog number M7240; IHC methodology described in Supplementary Methods). An experienced pathologist (NvG), blinded for treatment status and outcome, scored all IHC stainings. The percentage of tumor cells was determined on hematoxylin-eosin staining. The EGFR score was determined by the percentage of positive tumor cells multiplied by the staining intensity (0, 1, 2, or 3), and for Ki67 staining the percentage of tumor cells with positive nuclear staining was reported.

### Cetuximab and sEGFR concentration in blood

Serum samples for PK were collected before and directly after the first infusion, at day 6 (before [^89^Zr]Zr-cetuximab PET/CT) and before cycles 2 and 3. Serum cetuximab concentrations were evaluated using an ELISA kit (ABIN4886391; AffinityImmuno Inc, Charlottetown, PE, Canada) as per the manufacturer’s instructions.

Human plasma sEGFR was assessed in heparin blood collected at baseline and after 8 weeks of treatment. An ELISA development kit for human sEGFR was used (catalog number DEGFR0; R&D Systems) as per the manufacturer’s instructions (methodology for both ELISA procedures in Supplementary Methods).

### Statistics

Detailed statistical methods are included in the Supplementary Methods. Briefly, we used standard descriptive statistics to describe the study population, and used standard statistical tests where appropriate (e.g., Student’s *T*, Mann-Whitney’s *U*, and Fisher’s exact tests). Uni- and multivariable analyses for patient outcome (treatment benefit, PFS, OS) were performed with Firth’s penalized logistic and Cox regression analyses, using propensity score adjustment to account for potential confounders (age, gender, WHO performance status, number of metastases, *BRAF* mutation status, primary tumor sidedness, and standard versus escalated cetuximab dose), in all *RAS* wild-type patients and after restriction to *RAS* and *BRAF* wild-type patients with left-sided primary CRC. The area under the receiver operating characteristic curve (ROC AUC) was used to assess discrimination.

We used generalized linear mixed models with random intercepts to take clustering into account for analyses with multiple measurements per patient (and per lesion). To improve model fit, [^89^Zr]Zr-cetuximab SUV_peak_ and SUV_mean_, [^18^F]FDG SUV_peak_, tumor size, tumor perfusion, and EGFR IHC score were natural log-transformed (the latter after adding + 1 to each value as the IHC score contained zero’s). We used the marginal *R*^2^ to estimate the variance in the dependent variable explained by the fixed factor(s) in these models. To assess the explained variance for non-clustered data, we used regular linear regression models and the corresponding *R*^2^.

Data were analyzed with R version 3.2.1 for MAC OS. We report estimates together with 95% confidence intervals (CI). All *P* values are two-sided and not corrected for multiple testing.

## Results

### Patient characteristics

Between May 2014 and July 2017, 35 patients (median age of 64 years) with advanced *RAS* wild-type mCRC were enrolled, 31 in the image-guided dose escalation part and four in the exploratory low pre-dose part. The majority was male (71%), nine (26%) had a right-sided tumor, and four (12%) had a *BRAF* mutation (all right-sided). All patients received prior standard (combination) chemotherapy (Table 1).

**Table 1:** Patient characteristics according to study part and visual [^89^Zr]Zr-cetuximab assessment

	All patients	Image-guided dose escalation part		Exploratory low pre-dose part
		Visual [^89^Zr]Zr-cetuximab		
		Positive	Negative^a^	
Number of patients	35	21	10	4
Age (years), median (range)	64 (50–82)	65 (50–82)	61 (50–71)	67 (55–68)
Male gender, *n* (%)	25 (71.4)	15 (71.4)	8 (80.0)	2 (50.0)
ECOG performance status, *n* (%)				
0	9 (25.7)	5 (23.8)	4 (40.0)	0 (0.0)
1	23 (65.7)	14 (66.7)	5 (50.0)	4 (100.0)
2	3 (8.6)	2 (9.5)	1 (10.0)	0 (0.0)
Right-sided primary tumor, *n* (%)	9 (25.7)	8 (38.1)	0 (0.0)	1 (25.0)
*BRAF* mutation, *n* (%)	4 (11.8)	4 (20.0)	0 (0.0)	0 (0.0)
Unknown, *n*	1	1	0	0
Tumor load, median (range)				
Number of affected organs	3 (1–6)	3 (1–5)	2 (1–4)	2 (1–6)
Number of lesions	6 (1–15)	7 (1–15)	6 (1–13)	4 (2–11)
Number of extra-hepatic lesions	4 (1–9)	4 (1–9)	2 (1–9)	3 (2–8)
Previous treatments, *n* (%)				
Fluoropyrimidine	35 (100.0)	21 (100.0)	10 (100.0)	4 (100.0)
Oxaliplatin	35 (100.0)	21 (100.0)	10 (100.0)	4 (100.0)
Irinotecan	32 (91.4)	20 (95.2)	8 (80.0)	4 (100.0)
Bevacizumab	24 (68.6)	13 (61.9)	7 (70.0)	4 (100.0)
Sunitinib	1 (2.9)	1 (4.8)	0 (0.0)	0 (0.0)

^a^In this group eight patients underwent a dose escalation

### Visual [^89^Zr]Zr-cetuximab tumor uptake, cetuximab escalation, and clinical outcome

In the image-guided dose escalation part of the study, 21 (68%) patients had at least one extra-hepatic tumor lesion with visible uptake at [^89^Zr]Zr-cetuximab PET/CT with 500 mg/m^2^ pre-dose. Visual intra-patient heterogeneity was observed in 15 (54%) out of 28 patients with multiple extra-hepatic lesions. Of the 10 (32%) patients without reported tumor uptake, eight (80%) subsequently received an escalated dose of cetuximab: three from 500 mg/m^2^ to 750 mg/m^2^, three to 1000 mg/m^2^, and two to 1250 mg/m^2^. The remaining two patients did not undergo dose escalation due to logistic reasons (*n* = 1) or rapid progression (*n* = 1). No dose-limiting toxicity or dose reductions occurred after dose escalation in eight patients.

**Fig. 2:**
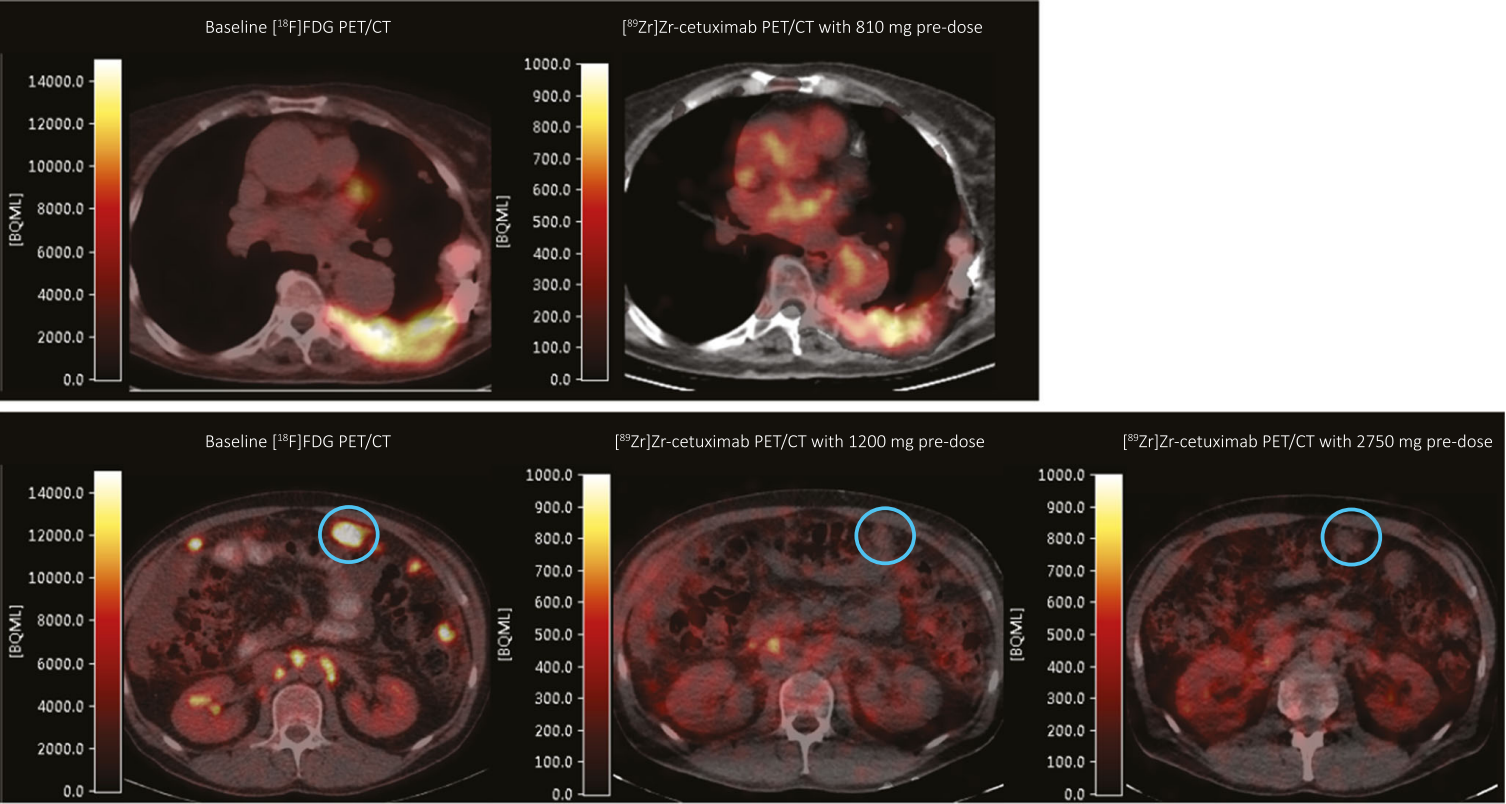
PET/CT fusion images of tumor uptake of two patients. The upper row illustrates a rib lesion on [^18^F]FDG PET/CT and tumor uptake on [^89^Zr]Zr-cetuximab PET/CT with a therapeutic pre-dose (500 mg/m^2^). The lower row depicts a patient with a peritoneal lesion (blue circle) without tumor uptake on [^89^Zr]Zr-cetuximab PET/CT with the therapeutic dose and escalated dose (1250 mg/m^2^)

None of the 8 patients with a negative [^89^Zr]Zr-cetuximab PET scan (500 mg/m^2^ pre-dose) demonstrated visual tumor uptake with an escalated cetuximab dose at the second scan (Fig. 2). In the exploratory low pre-dose part of the study none of the six extra-hepatic tumor lesions that were visually negative on the second 500 mg/m^2^ pre-dose scan was positive on the first lower 100 mg/m^2^ pre-dose (data from three of the four patients as one rapidly progressed before the second scan). Eight lesions were positive on both PET and CT.

After 8 weeks of cetuximab monotherapy, first CT evaluation demonstrated partial response in three patients, stable disease in 16, and progressive disease in 12, resulting in an overall treatment benefit of 61% (95%CI 42–78%). Follow-up continued through February 2019, at which time all patients had progressed and 30 of 31 patients had died (overall median PFS 4.2 months and OS 9.1 months).

Of the 21 patients with visible [^89^Zr]Zr-cetuximab uptake on the first scan (with subsequent standard cetuximab treatment), 52% had treatment benefit versus 80% of the 10 patients without visual uptake (and thus with an intention to dose escalate; *P* = 0.24). Similarly, the median PFS was 3.6 versus 5.7 months (log-rank *P* = 0.14; hazard ratio (HR) 1.74, 95%CI 0.82–4.00), and the median OS 7.1 versus 9.4 months (log-rank *P* = 0.27; HR 1.50, 95%CI 0.71–3.40; Figs. S2 and S3). Adjusting for age, gender, WHO status, and number of metastases did not substantially change these results, and additional exclusion of patients with primary right-sided or *BRAF*-mutated cancer—all from the [^89^Zr]Zr-cetuximab visual positive group and with a very poor outcome—resulted in an OR for PD at 8 weeks of 1.01, and HRs for PFS and OS of 0.99 and 0.78 (all *P* values > 0.65; Tables S1–3).

Combining data from both study parts, and accounting for potential confounders, no relation was observed between escalated and standard cetuximab dosing (OR for PD at 8 weeks 0.65, HRs for PFS and OS 0.76 and 0.79; all *P* values > 0.70; Tables S1–3).

### Quantitative [^89^Zr]Zr-cetuximab tumor uptake per lesion and clinical outcome

Combining data from all 35 patients we identified 138 extra-hepatic tumor lesions on [^89^Zr]Zr-cetuximab PET/CT with a 500 mg/m^2^ pre-dose. In total 83 lesions in 30 patients (median 2.5, range 1–7 lesions per patient) were sufficiently large (volume of tumors ≥ 4.2 mL) for quantitative assessment. Visual and quantitative assessment of [^89^Zr]Zr-cetuximab PET matched on a lesion level, as visually negative lesions had a lower geometric mean SUV_peak_ than visually positive lesions (2.5 versus 3.7, *P* = 0.003). However, the geometric mean SUV_peak_ was not associated with overall visual [^89^Zr]Zr-cetuximab PET scan positivity (geometric mean of 3.0 versus 3.5 for visually negative versus positive scans, *P* = 0.29), thus did not relate to the dose escalation indication (geometric mean of 2.9 and 3.5 for patients receiving escalated versus standard cetuximab dose, *P* = 0.18).

**Fig. 3:**
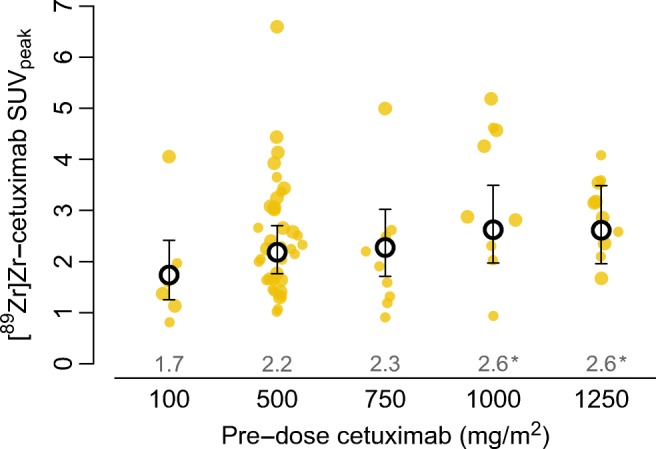
Relation between cetuximab pre-dose and SUV_peak_ in visually negative lesions from patients who underwent a second [^89^Zr]Zr-cetuximab PET/CT (11 patients, 36 lesions). Black circles and values above the *x*-axis show the geometric means at each pre-dose level and error bars show the corresponding 95% confidence interval estimated with a linear mixed regression model (using Satterthwaite’s approximations to degrees of freedom under restricted maximum likelihood). *P < 0.05 compared with the 100 mg/m^2^ pre-dose. *P* for trend, 0.014. Large dots represent lesions ≥ 4.2 mL and small dots < 4.2 mL (which were included in this analysis due to otherwise too limited number of lesions). All lesions remained visually negative irrespective of pre-dose

Comparing the repeat [^89^Zr]Zr-cetuximab PET scans quantitatively with pre-doses between 100 and 1250 mg/m^2^ showed that dose escalation increased [^89^Zr]Zr-cetuximab uptake in visually negative lesions in a dose-response manner with a trend of 7.8% (95%CI 1.5–14.6%; *P* = 0.014) increase in geometric mean SUV_peak_ per 250 mg/m^2^ increase in pre-dose (Fig. 3), without resulting in a change in visual assessment.

**Fig. 4:**
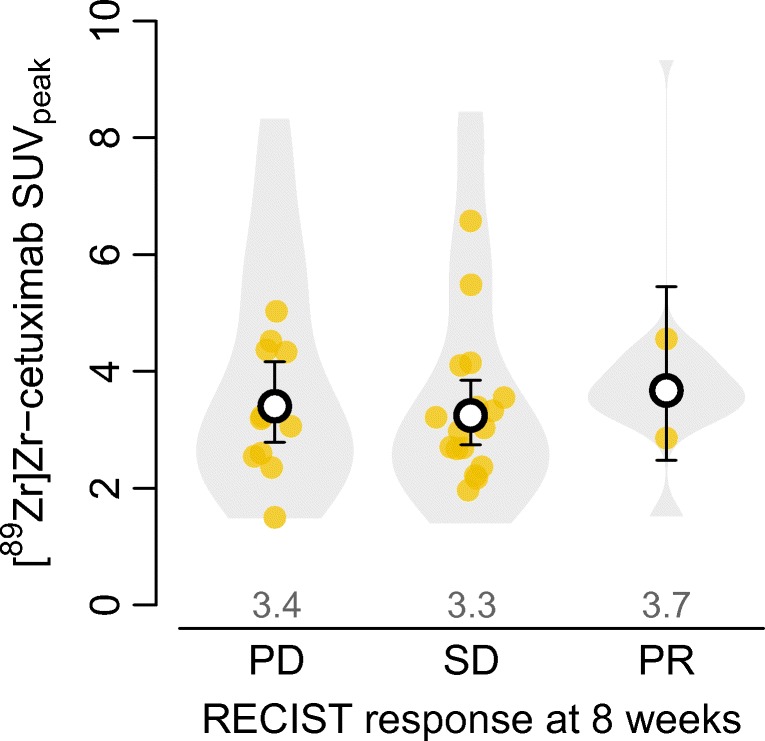
Violin plot of SUV_peak_ distribution across lesions according to best RECISTv1.1 response per patient after 8 weeks of treatment. Bottom and top 1% of SUV_peak_ values are truncated. Points show geometric mean uptake per patient. Black circles and values above the *x*-axis show the geometric mean SUV_peak_ of patients who had progressive disease (PD; 11 patients, 31 lesions), stable disease (SD; 17 patients, 43 lesions), or partial response (PR; 2 patients, 9 lesions) as estimated with a linear mixed regression model; error bars show the corresponding 95% confidence intervals. Two-sided Wald’s *P* values with Satterthwaite’s approximations to degrees of freedom under restricted maximum likelihood: *P* = 0.71 and *P* = 0.72 for SD respectively versus PR

There were no differences in geometric mean SUV_peak_ on [^89^Zr]Zr-cetuximab PET/CT with 500 mg/m^2^ pre-dose between patients with progressive disease, stable disease, and partial response at 8 weeks (Fig. 4). Adjusting this relation for treatment dose and lesion size did not change results (Table S4). On a lesion level, SUV_peak_ did also not correlate with CT-based lesion size change at 8 weeks (diameter changed on average − 5% (95%CI − 14 to 3%; *P* = 0.23) per twofold increase in SUV_peak_, which also remained unchanged after dose and lesion size adjustment).

The geometric mean SUV_peak_ per patient did not relate to PFS (HR per SD increase in SUV_peak_ was 0.69, 95%CI 0.43–1.09, *P* = 0.11). We did find an indication that geometric mean SUV_peak_ was positively related with OS after adjustment of potential confounders, particularly when restricting the analyses to left-sided *BRAF* wild-type cancer patients: HR per SD increase in SUV_peak_ was 0.56 (95%CI 0.32–0.95, *P* = 0.03) (Tables S2–3; corresponding survival curves are shown in Figs. S2–3). PFS and OS were neither related to the geometric mean nor related to the maximum SUV_peak_ per patient. Besides SUV_peak_, SUV_mean_ was evaluated per tumor lesion. SUV_mean_ was highly correlated to SUV_peak_. Expressing uptake in SUV_mean_ did not result in a relation between [^89^Zr]Zr-cetuximab PET/CT and clinical outcome (Fig. S4).

### Pharmacokinetics of cetuximab, [^89^Zr]Zr-cetuximab, and soluble EGFR

Mean C_max_, C_min_, and AUC are described in Table S5. AUC and C_max_ of the first cycle and C_min_ after the first and third cycles do not differ between patients with and without treatment benefit and did not correlate to PFS and OS. AUC and C_min_ cycle 1 was not correlated with tumor SUV_peak_ and SUV_mean_ on [^89^Zr]Zr-cetuximab PET/CT.

[^89^Zr]Zr-cetuximab plasma levels highly correlated with image-derived blood activity (*R*^2^ = 0.81, *P* < 0.001) and with unlabeled cetuximab concentration in serum (*R*^2^ = 0.54, *P* < 0.001, Fig. S5).

Pre-treatment plasma sEGFR levels ranged from 2.9 to 6.4 ng/mL for all patients, with higher sEGFR levels in patients with treatment benefit (mean 4.4 versus 3.8, *P* = 0.006; ROC AUC 0.75, 95%CI 0.58–0.91, Fig. S6). On-treatment sEGFR was higher for all patients (mean 4.2 versus 9.3 ng/mL, *P* < 0.001). The sEGFR level was not related to tumor EGFR expression (4.2 for ≤ 10% versus 4.2 ng/mL > 10% EGFR staining, *P* = 0.8), [^89^Zr]Zr-cetuximab blood concentration on PET, or %ID in the liver (a potential “sink” organ for cetuximab-sEGFR complexes; *P* = 0.33 and *P* = 0.62 respectively).

### EGFR expression and Ki67 staining of tumor tissue

From 26 of 30 included patients with tumors > 4.2 mL on [^89^Zr]Zr-cetuximab PET with 500 mg/m^2^ pre-dose, FFPE tumor material was available for IHC, in 13 patients this was resected material (lesion not available for evaluation on PET/CT) and in 13 patients it was biopsy material of a tumor lesion present at the start of treatment. A per-lesion comparison showed that SUV_peak_ on [^89^Zr]Zr-cetuximab PET of biopsied lesions increased 16% (95%CI 1 to 33%; *P* = 0.037) for each doubling in (EGFR + 1) score, with an *R*^2^ of 34% (Fig. S7A). Analyzing all 73 lesions (> 4.2 mL) from 26 patients showed that the geometric mean SUV_peak_ per patient increased by 4% (95%CI −2 to 11%; *P* = 0.17) per doubling in (EGFR + 1) score, assuming identical EGFR expression in all tumor lesions per patient (Fig. S7B). Tumor lesions with an EGFR score > 10 had a higher SUV_peak_ (mean SUV_peak_ 3.4 for ≤ 10 versus 6.8 for > 10 EGFR score, *P* = 0.012).

Proliferation, evaluated using Ki67 staining, did not correlate with [^89^Zr]Zr-cetuximab and [^18^F]FDG PET/CT results. There was no relation with treatment benefit (*P* = 0.74) or PFS (*P* = 0.16). There was a relation between OS and Ki67 staining, with a HR of 1.38 per 10% increase (95%CI 1.04–1.84, *P* = 0.02; Table S1–S3).

### Tumor perfusion

In 10 patients [^15^O]H_2_O PET/CT scans were performed to determine tumor perfusion. A total of 19 tumor lesions were evaluated, all sizes included. One patient only underwent a [^89^Zr]Zr-cetuximab PET/CT with 100 mg pre-dose and was off treatment due to rapid progression. On a lesion level [^89^Zr]Zr-cetuximab SUV_mean_ increased 33% (95%CI 12–58%; *P* = 0.0011) per doubling in mL/cm^3^/min perfusion (K1), with an *R*^2^ of 42% (Fig. S8).

### Lesion characteristics affecting [^89^Zr]Zr-cetuximab tumor uptake

Tumor volume (for lesions > 4.2 mL), location of tumor lesion (i.e., adrenal, bone, soft tissue, primary tumor, lung, lymph node, and peritoneal lesions in decreasing order for [^89^Zr]cetuximab uptake), and [^18^F]FDG uptake were univariably related with [^89^Zr]cetuximab uptake. The estimated variance in SUV_peak_ explained by location was 34%, 23% for size, and 10% for [^18^F]FDG uptake (Table S6 and Fig. S9).

Using multivariable mixed model analysis, the explained variance in [^89^Zr]cetuximab SUV_peak_ for tumor volume, location, and [^18^F]FDG uptake combined was 48%. Correction of the [^89^Zr]Zr-cetuximab uptake for size, location, and metabolic activity did not lead to a correlation between tumor uptake and anatomical change on CT scan after 8 weeks (adjusted change in diameter on average − 6% (95%CI − 17 to 5%) per twofold increase in SUV_peak_, *P* = 0.28). EGFR expression and tumor perfusion were not added in this multivariable analysis, as these data were only available for a limited number of lesions.

## Discussion

Treatment selection tools are needed to improve treatment efficacy in patients with solid tumors. In this image-guided dose escalation study we evaluated whether [^89^Zr]Zr-cetuximab PET/CT can be used to predict cetuximab monotherapy efficacy in patients with *RAS* wild-type metastatic colorectal cancer, based on the hypothesis that visible [^89^Zr]Zr-cetuximab uptake is a prerequisite for treatment response. There was no relationship between [^89^Zr]Zr-cetuximab PET positivity and treatment benefit or survival. Tumor uptake of [^89^Zr]Zr-cetuximab (as measured with SUV_peak_) did not correlate to changes in tumor size on CT, treatment benefit, nor PFS, neither at a patient level nor at a metastasis level. However, since patients without visual uptake on [^89^Zr]Zr-cetuximab PET/CT with the standard therapeutic pre-dose (500 mg/m^2^) had a dose escalation, treatment benefit of visually PET-negative patients could potentially be positively influenced. Yet, none of the second [^89^Zr]Zr-cetuximab PET/CT with a higher cetuximab pre-dose resulted in a visual tumor uptake. Also, the fact that the majority of the patients without visual tumor uptake had treatment benefit makes [^89^Zr]Zr-cetuximab PET/CT unsuitable as a predictive biomarker. It can be concluded that [^89^Zr]Zr-cetuximab PET/CT lacks ability to discriminate between insufficient/low and adequate dose levels required for anti-cancer effectiveness of cetuximab.

This study was designed based on previous work, in which 10 patients were scanned multiple times after injection with comparable amounts of [^89^Zr]Zr-cetuximab tracer and a therapeutic pre-dose of cetuximab (500 mg/m^2^). These results showed a positive predictive value of 67% and negative predictive value of 75% (based on the PET scan time interval of 6 days), resulting in a potentially promising relation between PET uptake and response. However, one of four responding patients lacked uptake. In light of the current data, we conclude that due to chance and the small sample size the results from the pilot study could not be confirmed. We did find an indication that the geometric mean SUV_peak_ per patient may be related to OS in a small subgroup of only left-sided *BRAF* wild-type cancer patients, adjusted for potential confounders. This potential relation could be a result of multiple testing or the dose escalation (less likely as there was no relation with PFS). Even if the relation is correct, it cannot be used to differentiate between patients with and without treatment benefit.

The [^89^Zr]Zr-cetuximab PET/CTs were performed with a pre-dose (500 mg/m^2^) to determine the percentage of the therapeutic dose in the tumor. Pre-dosing has been successfully applied for imaging of radioimmunotherapy [18], antibody-drug conjugates [19], and diagnostics [20], to increase tumor targeting of radiolabeled antibodies by blocking nonmalignant binding sites. A previous study, using SPECT, demonstrated that tracer dose of anti-EGFR mAbs without pre-dose was almost entirely absorbed by the liver, hampering tumor visualization [21]. In our study, escalating the therapeutic pre-dose and performing a second [^89^Zr]Zr-cetuximab PET/CT in patients with an initially negative PET/CT did not result in visibly increased tumor uptake. Quantitatively, a small increase in average tumor SUV_peak_ was demonstrated. This was most likely a result of an increased background of retained tracer 6 days p.i. due to percentage-wise less biological excretion (Fig. S10). A potential drawback of a therapeutic pre-dose, which is approximately 100 times higher than the tracer dose, is that it can partially or irregularly saturate the therapeutic target on the tumor cells [22], in this case EGFR. Yet, we previously showed slow PK and initially reversible EGFR binding resulting in a homogeneous distribution of unlabeled and labeled cetuximab, with an interval of ≤ 2 h between the two administrations [11]. To explore potential target saturation, a [^89^Zr]Zr-cetuximab PET/CT was performed with a pre-dose of 100 mg and 500 mg/m^2^ cetuximab in three patients. Visual and quantitative results of the PET/CT with a pre-dose of 100 mg were not relevantly different with a pre-dose of 500 mg/m^2^ unlabeled cetuximab. (Fig. S2). We conclude that it is unlikely that pre-dose relevantly changes [^89^Zr]Zr-cetuximab PET/CT results, or explain the absence of a correlation with clinical response data. However, this is based on a small explorative cohort.

Response and survival data for patients treated with the standard versus escalated cetuximab dose were comparable in this small cohort. There is a beneficiary trend in the dose escalation group; this results from imbalanced prognostic characteristics. In the intention to escalate group there are less patients with right-sided disease and no patients with *BRAF*-mutated tumors (Table 1). Currently, patients with these tumor characteristics do not receive anti-EGFR mAbs due to lack of efficacy. Correcting for these factors resulted in comparable response and survival data (Tables S1–S3). If an increased dose results in more treatment benefit remains unknown. Some clinical studies reported an association between higher global clearance and lower trough levels of cetuximab and a shorter PFS [8, 9], whereas others did not [23, 24]. All studies were limited given the less than 100 patients included. In this study drug exposure measured in PK samples did not correlate with treatment efficacy, regardless of dose (Table S4). In a larger clinical study, dose escalation based on absent skin toxicity resulted in an increased response rate, but did not improve PFS or OS [25]. Still, even if a higher dose cetuximab improved outcome, [^89^Zr]Zr-cetuximab PET/CT remained visually negative with the higher pre-dose and thus cannot discriminate between patients that do and do not benefit from cetuximab monotherapy.

This study reports large differences in tumor [^89^Zr]Zr-cetuximab uptake within and between patients [11, 12], without a relation with treatment benefit of cetuximab. Understanding factors contributing to the imaging signal is important for the interpretation of [^89^Zr]Zr-cetuximab PET/CT imaging results, and relevant for future immuno-PET imaging studies. In this study, target expression (in this case EGFR) and the amount of shedded receptor (e.g., sEGFR), tumor size, location, drug (e.g., cetuximab) PK, perfusion, and metabolic activity were evaluated as factors that could affect [^89^Zr]Zr-cetuximab tumor accumulation.

EGFR expression determined with IHC was correlated to SUV_peak_ on [^89^Zr]Zr-cetuximab PET when evaluated per lesion. On a patient level, EGFR expression did not correlate to the geometric mean SUV_peak_ of all tumor lesions (> 4.2 mL). Others demonstrated that [^89^Zr]Zr-cetuximab tumor uptake in patients with advanced head and neck cancer did not correlate with EGFR expression in the tumor as a continuous variable, but SUV and tumor to background ratio were significantly higher in patients with a high versus low EGFR score [26]. These findings are supported by preclinical data, which demonstrated that [^89^Zr]Zr-cetuximab tumor uptake not only is dependent on EGFR expression but also is influenced by other pharmacokinetic and dynamic mechanisms [10].

Tumor size correlated with [^89^Zr]Zr-cetuximab tumor uptake, even after exclusion of small lesions that are affected by partial volume effects. We hypothesize that aggressively growing lesions are larger at the time of detection and that aggressiveness is related to higher EGFR expression [27] as well as higher [^18^F]FDG uptake [28]. Additionally, larger lesions have a better developed vascular structure as they cannot rely on diffusion alone [29]. Indeed, tumor perfusion evaluated on [^15^O]H_2_O PET was positively correlated with [^89^Zr]Zr-cetuximab tumor uptake (Fig. S8). Thus, more efficient drug delivery results in more uptake of radiolabeled antibody. To our knowledge, this is the first clinical study in which a relation between tumor perfusion and uptake of radiolabeled antibodies is found in humans.

The location of the tumor lesion was also related to [^89^Zr]Zr-cetuximab tumor uptake. Possible explanations are that differences between sites occur due to biological factors, such as differences in organ perfusion or EGFR expression, and technical factors related to PET quantitation, such as assessment of background and lesional size. For instance, lymph node lesions are generally smaller than primary tumors, and lung lesions have a lower background compared with adrenal gland lesions which are located next to the liver, or primary tumors that can have interference of fecal matter containing excreted metabolites of [^89^Zr]Zr-cetuximab. Bone seeking properties of [^89^Zr]Zr-labeled mAbs could add to the high uptake in bone; however, no relative increase in background activity in normal bone was observed. In a multivariate analysis, indeed, 48% of the variance of [^89^Zr]Zr-cetuximab uptake was explained by tumor location, size, and [^18^F]FDG uptake. However, correction for these factors did not improve the relation of [^89^Zr]Zr-cetuximab uptake with response on CT. Of note, EGFR expression and tumor perfusion were not added to these analyses, as these data were only available for a limited number of lesions. However, as the level of explained variance is very high, it is recommended for future immuno-PET imaging studies to take target expression, tumor size, and location as well as metabolic volume and perfusion of tumor lesions into account. These factors affect specific and non-specific tracer uptake. Identifying these factors could give more insight into drug targeting and biodistribution. Correction for non-specific factors could potentially clarify discrepancies between quantitative [^89^Zr]Zr-cetuximab PET data and clinical outcome.

In plasma, sEGFR was assessed to evaluate its effects on [^89^Zr]Zr-cetuximab tumor uptake, as well as PK of cetuximab and [^89^Zr]Zr-cetuximab [12]. Soluble EGFR is the cleaved or transcribed extracellular part of EGFR and can bind cetuximab in circulation. Preclinically, sEGFR captures [^89^Zr]Zr-labeled antibody and redirects it to the liver, thereby decreasing tumor uptake [12]. There was no relation between baseline sEGFR and tumor targeting, %ID in liver, or concentration cetuximab and [^89^Zr]Zr-cetuximab in blood. This might be due to the extreme overdose of administered cetuximab compared with the measured sEGFR, which is about 78,000 times higher. Interestingly, baseline sEGFR was higher in patients who experienced treatment benefit compared with patients without benefit. Additionally, sEGFR is positively correlated with PFS and OS. Possibly, sEGFR has a negative feedback mechanism to inhibit cell growth via EGFR by capturing the natural ligands of EGFR and engaging in inactive dimers with transmembrane EGFRs [30]. One can envision that tumors with EGFR-driven growth and cell survival would have more sEGFR and would be sensitive to anti-EGFR mAbs. In small subsets, sEGFR has been evaluated as biomarker in different tumor types [31], but we are the first to show a positive correlation with treatment benefit and survival in patients with mCRC treated with cetuximab monotherapy.

### Conclusion

[^89^Zr]Zr-cetuximab PET/CT did not predict treatment benefit or PFS in patients with *RAS* wild-type mCRC treated with cetuximab monotherapy. Increasing cetuximab dose in patients with negative [^89^Zr]Zr-cetuximab PET/CT did not lead to visual tumor uptake or improved outcome. Tumor size, location, perfusion, and metabolic activity correlated with [^89^Zr]Zr-cetuximab uptake. *BRAF V600E* mutations, right-sidedness of the primary tumor, and low sEGFR are correlated with intrinsic resistance to cetuximab monotherapy.
